# Medical Education

**Published:** 1901-06-22

**Authors:** 


### The Hospital

A Journal of

The Medical Sciences and Hospital Administration.

Vol. XXX.—No. 769.] Edited by Sir Henry Burdett, K.C.B., and Solomon C. Smith, M.D., M.R.C.P. [June 22, 1901.

### Medical Education.

The question which has arisen between the General Medical Council and some of its constituent corporations, with regard to the manner in which the obligations of the first year of the educational curriculum must be fulfilled, although it has for the time been left in suspense by a reference to the Education Committee of the Council, none the less calls for the consideration of all who are interested in the welfare of the profession. The matter in dispute is really vital; and the reference to the Committee only provides for the postponement of a struggle which must be forced upon the Council before long, or which can only be avoided by an abdication of the position which the legislature intended it to fill. Such postponements are seldom serviceable to either of the parties to a dispute; and although, on the present occasion, the course adopted was partly due to a desire to abbreviate the session in order that some members of the Council might take part in the celebration of the ninth jubilee of the University of Glasgow, it would probably have been wiser to come to an immediate vote on the "sufficiency" of the regulations which the English Conjoint Board proposes for the future to enforce. Such a vote must inevitably be taken in November, and the necessity places the Council on the horns of a very uncomfortable dilemma. If it should strike its colours it will not only surrender every pretence of exercising authority over the conduct of medical education, but also the chief ground on which its continued existence can be justified; while, if it should nail its colours to the mast, it will have no choice but to bring the Conjoint Board and its constituent colleges before the Privy Council, with a very conceivable probability of being vanquished in the encounter.

The shortcomings of the Medical Council and its admitted failure to bring about the improvements in medical education which were expected from it, have been largely due to its composition, but partly, also, to its unwieldy magnitude and to the imperfect character of its authority. If it had been limited to twelve men, all nominated by the Crown and empowered to frame and issue regulations to which examining bodies would have been required to conform, there can be no doubt that both the standard of professional knowledge and the character of the training by which that knowledge was acquired, would by this time have undergone important changes. It will probably be necessary to fall back upon some arrangement of this kind, whenever (if ever) politicians are able to withdraw their attention from squabbles for place and to take thought for the sanitary requirements of the nation. In the meanwhile, everyone who is acquainted with the facts must sorrowfully admit that the education of the medical profession has not been brought up to the level of the scientific knowledge of the day, and is not so conducted as to secure, if we may parody a time-honoured formula, the greatest proficiency of the greatest number. Individuals supply its deficiencies by the force of industry; sometimes, it may be, by the force of genius; but it cannot be contended that the average young man is prepared or tested in the best possible way for the discharge of the duties which he proposes to undertake, and upon his fulfilment of which the lives and fortunes of his patients may come to depend. Nor can it be said that the method of selection is much better than that of preparation. Every examiner is acquainted with types which display an absolute lack of some one or more of the qualities essential to the proper fulfilment of medical or surgical duties. The candidate for a surgical license who was shown a case of talipes varus in an infant a month old, and who, when told what it was, and asked how it should be treated, replied that he would "give tonics, and not suffer the child to put its feet to the ground," should have been bowed out of the examination room, not for that occasion only, but for ever. The practitioner who prescribed "Mist. Sodæ Co." for intestinal obstruction with stercoraceous vomiting, and who said at the inquest that he "looked upon it as a case of dyspepsia," was one whose examiners could hardly be acquitted of criminal negligence.

The particular question now at issue between the Council and the Conjoint Board is whether the last year of education at an ordinary school in which some instruction is given in the rudiments of physical science can be accepted as the first of the five years of the medical curriculum, the year in which physical science is supposed to be the main subject of study. To maintain the affirmative is obviously to strike off the first year of the medical curriculum by a sidewind, and there can be little doubt that this is the real object of the colleges, it having been found that the requirement of five years has diminished the number of candidates. It is obvious that if the colleges, in defiance of the Council, can strike off the first year, they can strike off the second if they please, and may compete in paring down the curriculum. In these circumstances it is to be hoped that the Council will maintain a position which is equally important for the profession and for the public.

